# Nasal Irrigation with Licorice Extract for Allergic Rhinitis: A Clinical Study Evaluated by Subjective Assessments and Meridian Electrical Conductance

**DOI:** 10.3390/life15111667

**Published:** 2025-10-25

**Authors:** Pei-Rung Yang, Yung-Hsiang Chen, Chao-Yang Chang, Bo-Cheng Rau, Yu-Ching Cheng, Yao-Hsu Yang, Ching-Yuan Wu, Geng-He Chang

**Affiliations:** 1 Department of Traditional Chinese Medicine, Chang Gung Memorial Hospital, Chiayi 613, Taiwan; 2 School of Traditional Chinese Medicine, College of Medicine, Chang Gung University, Taoyuan 333, Taiwan; 3 Graduate Institute of Integrated Medicine, College of Medicine, China Medical University, Taichung 404, Taiwan; yhchen@mail.cmu.edu.tw; 4 School of Medicine, College of Medicine, China Medical University, Taichung 404, Taiwan; 5 Department of Otolaryngology—Head and Neck Surgery, Chang Gung Memorial Hospital, Chiayi 613, Taiwan; 6 Faculty of Medicine, College of Medicine, Chang Gung University, Taoyuan 333, Taiwan; 7 Graduate Institute of Clinical Medical Sciences, College of Medicine, Chang Gung University, Taoyuan 333, Taiwan; 8 Head and Neck Infection Treatment Center, Chang Gung Memorial Hospital, Chiayi 613, Taiwan

**Keywords:** rhinitis, nasal irrigation, Glycyrrhiza, licorice, herbal, meridian, electrical conductance

## Abstract

**Objective**: Allergic rhinitis (AR) continues to adversely affect the life quality of a substantial patient population, highlighting the necessity for enhanced treatment modalities. Our research utilized licorice extract (LE) in nasal irrigation for managing this condition, with its therapeutic efficacy gauged against traditional saline nasal irrigation (SNI) through clinical trials. Additionally, the study incorporated traditional Chinese medicine (TCM) principles, measuring not just subjective symptom relief but also the objective shifts in lung meridian electrical conductance (MEC), to provide a comprehensive evaluation of the treatment’s effectiveness. **Methods**: Based on our previous laboratory and animal studies, we developed an LE solution and applied it through nasal irrigation to treat AR. In a one-month controlled trial, 60 patients with AR received either licorice nasal irrigation (LNI) or SNI daily. We assessed treatment efficacy by subjective questionnaire scores (Total Nasal Symptom Score [TNSS] and 22-item Sino-Nasal Outcome Test [SNOT-22]) and objective lung MEC analysis. **Result**: In the trial, 30 participants were randomly allocated to each group, and 28 individuals in the LNI group and 24 in the SNI group finished the study without any side effects. The LNI group had better improvements in sneezing, nasal itchiness, and rhinorrhea, along with a greater overall TNSS reduction. On the SNOT-22, the LNI group scored better across most nasal and extra-nasal symptoms, sleep, and physiological and psychosocial well-being. Participants were sorted into low, normal, and high lung MEC subgroups. After treatment, those in the LNI group normalized their lung MEC levels in both the low and high subgroups, which was not observed in the SNI group. **Conclusions**: LNI markedly improves symptoms in patients with AR, enhancing their quality of life. This treatment method, integrating Western and TCM practices, also normalizes abnormal lung MEC values following therapy. It offers a method of objectively validating the effectiveness of treatments based on TCM theories.

## 1. Introduction

Allergic rhinitis (AR) is a prevalent chronic condition across various global regions, significantly impacting the quality of life of numerous individuals. The absence of timely and appropriate therapeutic intervention may lead to complications, notably sinusitis and asthma [1]. Despite the availability of a range of pharmacological interventions, including oral antihistamines, steroid nasal sprays, and mast cell stabilizers [2,3], along with various nasal surgical procedures, the domain of AR management still necessitates concerted efforts for enhancement.

The practice of nasal irrigation using saline has been highlighted as an effective additional therapy in several guidelines for managing chronic rhinitis [4]. Numerous studies have explored adding various substances to saline, such as steroids and xylitol [5,6], showing a potential improvement in relieving the symptoms of chronic rhinitis compared to using saline alone. Additionally, we have worked on developing a licorice extract (LE) for treating AR through nasal irrigation [7,8]. Licorice, a traditional medicinal herb widely used in Chinese medicine, and described in the Chinese pharmacopoeia as comprising three species—*Glycyrrhiza uralensis*, *Glycyrrhiza inflata*, and *Glycyrrhiza glabra,* is known for its anti-inflammatory [9], anti-allergic [10], and immune-modulatory effects [11], with glycyrrhizic acid (GA) generally considered its main bioactive compound, while *Glycyrrhiza glabra* is the species used in this study. This new formulation has shown promising results, notably in suppressing mast cells to release histamines, a finding supported by both cellular and animal experiments [7].

The meridian theory is a fundamental concept in traditional Chinese medicine (TCM), integral to the localization of acupuncture treatments [12]. Generally, allergic rhinitis is posited to be associated with the dysfunction of the lung meridian [13]. Some research has corroborated that the meridians in TCM correspond to the pathways of nerves in the human body [14]. Consequently, a device, meridian energy analysis device (MEAD), designed to measure the skin electronic resistance across various meridians via microcurrent has been developed to objectively assess the state of a patient’s meridians [15,16]. This device is now widely employed in contemporary Chinese medicine clinical practice [12,14,17,18].

Historically, there have been no investigations into the relationship between alterations in lung meridian and the extent of subjective symptomatic improvement in patients with allergic rhinitis pre- and post-treatment. Furthermore, the novel treatment of licorice nasal irrigation (LNI), devised by us previously, which represents an innovative approach within Chinese medicine, did not employ TCM assessment tools to analyze the variations in patients’ meridian status before and after treatment. Hence, we initiated this prospective study to examine the changes and correlation between the subjective symptom level and the electronic conductance value of the lung meridian in patients with AR after treatment, under the administration of either licorice or saline nasal irrigation (SNI), respectively.

## 2. Materials and Methods

### 2.1. Participants

The prospective, non-blinded, open label clinical study received approval from the Chang Gung Medical Foundation’s Institutional Review Board (approval number 201801970A3C501). It was also registered with ClinicalTrials.gov, bearing the identifier NCT04046393.

The sample size for our study was calculated using G*Power software 3.1 to detect significant differences with a statistical power of 80%, an effect size (Cohen’s d) of 0.8, and a type I error rate of 5% in a two-tailed test. To account for a potential dropout rate of 20%, we planned to enroll 30 participants in each of the control and experimental groups.

All participants provided their written informed consent after receiving a comprehensive explanation of the study’s purpose and procedures. Individuals diagnosed with AR and meeting the inclusion criteria were subjected to blood tests, clinical symptom evaluation, and naso-endoscopy examinations. These procedures were performed by a skilled otolaryngologist at the Chiayi Chang Gung Memorial Hospital’s Department of Otolaryngology.

The inclusion criteria for the study were specified as follows: (1) Patients aged 20 years or older who are willing to sign an informed consent form. (2) Participants are required to undergo a blood test showing a total IgE level greater than 120 IU/mL and a positive reaction to at least one inhaled allergen, such as dust mites. (3) Participants must not have used oral or nasal antihistamines or oral or nasal steroid sprays. If they have used these medications, they must have discontinued them at least two weeks before the study to eliminate any drug effects.

The exclusion criteria for the study were established as follows: (1) Women who are pregnant or breastfeeding are not eligible. (2) Individuals with a severe nasal septum deviation are excluded. (3) Patients must not have nasal polyps or sinusitis as determined by endoscopic examination. (4) Patients with a history of treatment for malignant tumors in the nasal cavity or nasopharynx are excluded from the study.

### 2.2. Licorice Solution for Nasal Irrigation

We created the LE following a standardized method established by previous research [7]. We placed 450 g of sliced licorice root (species: *Glycyrrhiza glabra*) into a 2 L pressure cooker and added 1100 mL of reverse osmosis purified water. The mixture was heated at 1.5 atmospheres and 120 degrees Celsius for 30 min. After cooking, we filtered the solution through a fine mesh to remove impurities and packaged it into 100 mL aliquots. To ensure consistency across batches, we measured the concentration of GA in the extract using high-performance liquid chromatography (HPLC).

HPLC analysis was conducted using an Agilent 1100 series system equipped with a Discovery^®^ C18 column (15 cm × 4.6 mm, 5 µm) (Agilent Technologies, Inc., Santa Clara, CA, USA). The reference compound, GA (ChromaDex, Los Angeles, CA, USA), exhibited a purity of 98.3% as determined by testing. The mobile phase consisted of 100% acetonitrile and 2% acetic acid in a 36% to 64% ratio, respectively. The flow rate was set at 0.6 mL/min, and the column temperature was maintained at 25 °C. Detection was performed using ultraviolet light within the 250–360 nm wavelength range to obtain the absorption spectrum.

### 2.3. Study Design

In this study, we anticipated enrolling sixty participants. They were randomly assigned to one of two treatments: LNI or SNI. Each patient received a high-capacity (300 mL), low-pressure squeeze bottle for the irrigation procedure. They were instructed to use the device daily for one month, following detailed guidelines provided to them.

**Figure 1:**
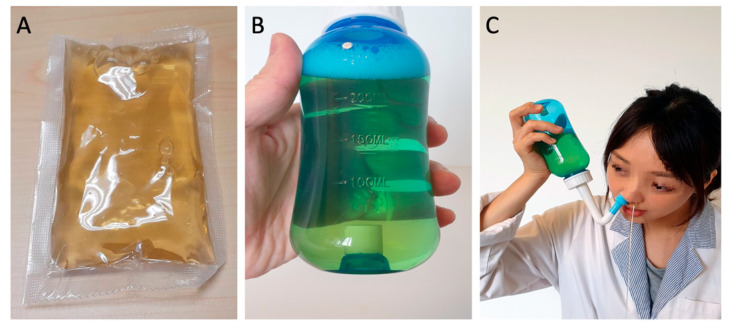
**Licorice nasal irrigation.** (**A**) Prepared licorice extract, 100 mL per pack; (**B**) licorice solution in a squeeze bottle (100 mL extract + 200 mL water + 3 gm non-iodized salt); (**C**) nasal irrigation demonstration.

For the LNI group, participants were given a daily packet of prepared LE each containing 100 mL. They were to add this solution to the squeeze bottle, followed by 200 mL of 37 °C drinking water and 3 g of non-iodized salt, mixing the solution well before nasal irrigation. The SNI group participants used a simpler mixture consisting of 300 mL of 37 °C drinking water and 3 g of non-iodized salt in the squeeze bottle, which was also well mixed before use for nasal irrigation (Figure 1).

Before starting treatment, an initial health assessment and interview were conducted for each participant. This included a physical examination and a detailed discussion about their medical history, lifestyle habits, and any medications they were currently taking. Participants who met the study criteria were subjected to subjective and objective treatment assessments at two- and four-weeks post-treatment initiation intervals. Furthermore, any adverse effects reported by participants during the clinical study were carefully recorded.

### 2.4. Primary Outcome Measures

The primary outcomes measured in this study were the changes in rhinitis-related symptoms following nasal irrigation. We used the Total Nasal Symptom Score (TNSS) and the 22-item Sino-Nasal Outcome Test (SNOT-22) for this purpose. The TNSS evaluates four main nasal symptoms: nasal blockage, rhinorrhea, sneezing, and nasal pruritus. Each symptom is rated on a scale from 0 to 3, with 0 indicating no symptoms, 1 for mild symptoms, 2 for moderate symptoms, and 3 for severe symptoms, making the total TNSS score range from 0 to 12.

The SNOT-22 is a validated questionnaire comprising 22 rhinitis-related items, including nasal symptoms, extra-nasal symptoms, sleep quality, physical and psychological status, that assesses the impact of nasal and sinus symptoms on quality of life. Each item is scored from 0 to 5, with 0 meaning no symptoms, 1 for mild, 2 for mild to moderate, 3 for moderate, 4 for severe, and 5 for as bad as it can be, leading to a total score range from 0 to 110 on the SNOT-22.

### 2.5. Secondary Outcome Measures

In this study, the secondary outcome was assessed through the electrical conductivity measurements of the lung meridian, where the standard meridian electrical conductance (MEC) is benchmarked between 30 and 60 microamperes [16]. Deviations from this standard indicate an abnormal state of neural electrical flow in the meridian. To ensure the integrity of measurements, all metallic objects were removed, and participants lay in a controlled environment with a temperature of 25 °C and humidity of 55% after a 15 min relaxation period.

**Figure 2:**
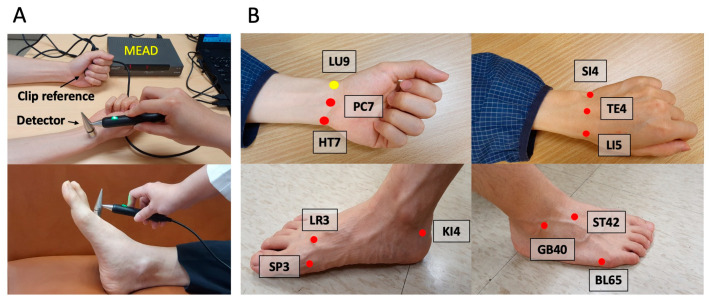
**Meridian energy analysis device (MEAD).** (**A**) MEAD with a clip reference and a detector; (**B**) 12 representing principal acupoints for the meridians: LU9 (Taiyuan acupoint for lung); PC7 (Daling acupoint for pericardium); HT7 (Shenmen acupoint for heart); LR3 (Taichong acupoint for liver); SP3 (Taibai acupoint for spleen); KI4 (Dazhong acupoint for kidney); SI4 (Wangu acupoint for small Intestine); TE4 (Yangchi acupoint for triple energizer); LI5 (Hegu acupoint for large intestine); GB40 (Qiuxu acupoint for gallbladder); ST42 (Chongyang acupoint for stomach); BL65 (Jinggu acupoint for urinary bladder). The number on the initials represents the number of acupoints on the meridian.

The study employed the Meridian Energy Analysis Device (MEAD; Me-100, MedPex Enterprises, Taiwan), designed in line with Ryodoraku theory, to measure the conductance of representative acupoints for each meridian [19]. The MEAD analyzed the electrical conductivity at these principal acupoints across the 12 meridians, ensuring a comprehensive evaluation of each meridian’s health. Participants engaged in the assessment by holding a metal cylinder in their left hand while a technician used a saline-dampened, pressure-sensitive probe to measure the specific points. Each acupoint measurement was taken thrice for accuracy, and the average was recorded, reflecting the meridians’ status and associated the organs’ condition (Figure 2). Several studies corroborated MEAD’s consistency and efficacy, evidenced by a high reproducibility rate of 93.2% [20]. The electrical conductance values of the lung meridian were specifically used for the objective evaluation of nasal irrigation efficacy in this research.

### 2.6. Statistical Analysis

The study’s data was analyzed with Microsoft Excel and MedCalc software 23.3.7. Significance was assigned at a *p*-value of less than 0.05. Changes within groups were analyzed using paired *t*-tests, while differences between the control and experimental groups were examined using unpaired *t*-tests. Chi-square and *t*-tests evaluated baseline group characteristics for comparability. Pearson’s correlation analyses were conducted to investigate the association between changes in subjective symptoms and changes in objective MEC measurements.

## 3. Results

### 3.1. Study Population

This research commenced at the outset of 2019 and spanned four years, encompassing data collection and completion. The initial patient enrollment process took place in an otolaryngology outpatient clinic. The otolaryngologist meticulously screened potential participants through a series of evaluations, including consultations, physical examinations, and blood tests. Only those meeting our stringent inclusion criteria were considered for the study.

**Figure 3:**
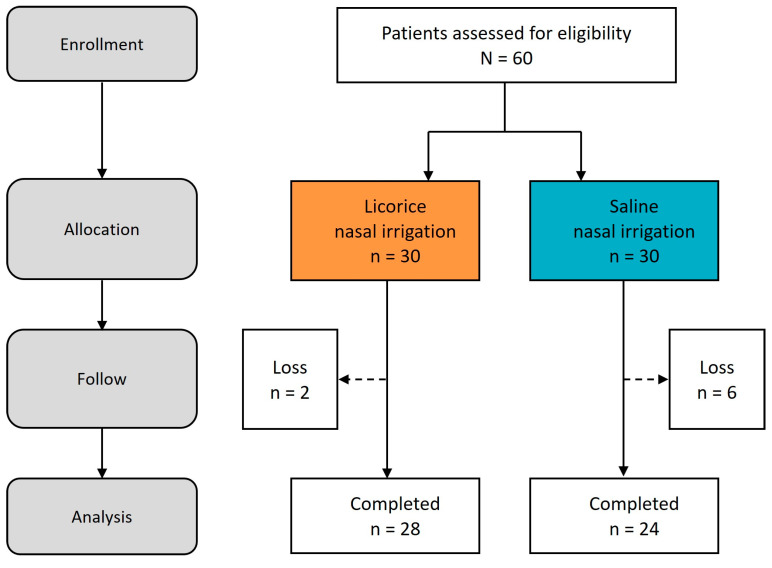
**Study process.** Flow diagram.

For this investigation, we enrolled a total of 60 patients, who were then randomly allocated to one of two treatment arms: LNI or SNI via a lottery system. It is noteworthy that two participants in the LNI group and six in the SNI group did not return for follow-up assessments due to personal reasons. Ultimately, the trial concluded with 28 patients in the LNI group and 24 in the SNI group successfully completing the study (Figure 3).

### 3.2. Demographic Characteristics

Table 1 details the demographic and clinical characteristics contrasting the two cohorts of patients with AR. Both groups predominantly consisted of females, with a representation of 60.7% in the LNI group and 75% in the SNI group. The average age was marginally higher in the LNI group (37.0 ± 11.7 years) compared to the SNI group (36.8 ± 9.7 years), with a *p*-value of 0.037. The IgE levels were comparable between the groups (126.4 ± 142.7 IU/mL for LNI vs. 97.6 ± 94.9 IU/mL for SNI, *p* = 0.271). Regarding medical comorbidities, a higher incidence of atopic dermatitis was observed in the SNI group (3.5% for LNI vs. 20.8% for SNI), although the difference did not reach statistical significance (*p* = 0.132). No significant differences were noted in the prevalence of asthma, GERD, or DM between the groups. Furthermore, all participants were non-smokers.

### 3.3. Glycyrrhizic Acid Concentration

**Figure 4:**
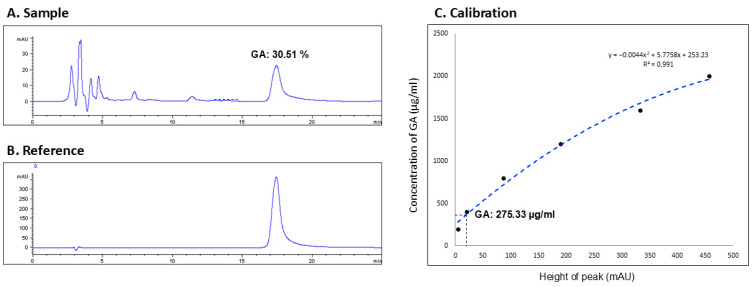
**High performance liquid chromatography (HPLC).** (**A**) Sample (licorice extract): calculating the glycyrrhizic acid [GA] proportion; (**B**) reference: GA compound; (**C**) calibration: calculating the GA concentration according to the equation.

In this study, the concentration of GA—a key active constituent of licorice—was quantified in the licorice preparation for nasal irrigation utilizing protocols established by preceding research [7]. The HPLC analysis of the LE identified nine distinct peaks (Figure 4A). A comparison with the GA standard (Figure 4B) revealed the presence of GA at the 17.43 min mark, corresponding to the ninth peak. Utilizing the integral method for analysis, GA constituted 30.51% of the combined peak areas (Figure 4A).

Subsequently, a series of GA concentrations (50, 100, …, 450, 500 mAU) were assayed to generate a calibration curve, yielding the equation y = −0.0044 *x*^2^ + 5.7758 *x* + 253.23, with a coefficient of determination *R*^2^ = 0.991 (Figure 4C). The calibration curve was then applied to determine the GA concentration in the licorice solution used for nasal irrigation, using the peak height (mAU) from the sample’s HPLC profile. This calculation indicated that the concentration of GA in the licorice solution was 275.33 μg/mL.

### 3.4. Primary Outcomes

#### 3.4.1. SNOT-22

Table 2 illustrates the changes in the SNOT-22 scores, assessing a range of symptoms and quality of life factors in patients with AR, before and after a month of treatment with two different nasal irrigation solutions. The assessment included categories such as nasal and extra-nasal symptoms, sleep quality, and both physical and psychological status.

Regarding nasal symptoms, the LNI showed significant improvement across all six evaluated symptoms, whereas SNI improved five, with olfaction being the exception where no notable improvement was recorded. In the context of extra-nasal symptoms, LNI was effective in ameliorating five out of six symptoms, with facial pain or pressure being the exception. On the other hand, SNI only marked a significant improvement in the symptoms of postnasal drip.

When considering sleep quality, patients treated with LNI experienced improvements in half of the assessed symptoms, whereas SNI did not significantly enhance sleep in any of the four categories. In evaluating physical status, all three aspects showed improvement with LNI, in contrast to SNI which did not exhibit significant enhancement. Similarly, LNI improved two out of three mental status measures, with no improvements noted with SNI.

Overall, LNI demonstrated significant improvement in 18 out of the 22 items on the SNOT-22, compared to SNI which improved six items. The aggregate SNOT-22 score decreased substantially from 36.07 ± 15.82 to 19.46 ± 11.97 (*p* < 0.0001) with LNI treatment, while SNI treatment resulted in a reduction from 25.92 ± 14.88 to 20.21 ± 15.43 (*p* = 0.0065).

#### 3.4.2. TNSS

**Figure 5:**
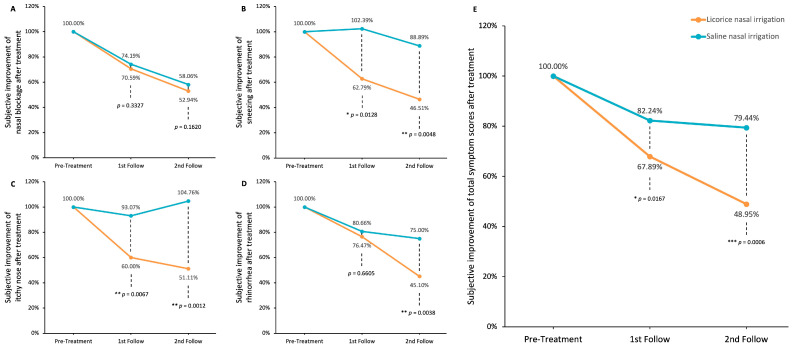
**Improvements in rhinitis-associated symptoms after LNI or SNI treatment.** (**A**) Nasal blockage; (**B**) sneezing; (**C**) itchy nose; (**D**) rhinorrhea; (**E**) total nasal symptom scores (TNSS). Data were analyzed using unpaired *t*-tests to compare LNI and SNI groups (* *p* < 0.05; ** *p* < 0.01; *** *p* < 0.0001).

Figure 5 delineates the alterations in TNSS subsequent to LNI and SNI at the two-week and four-week marks. The original symptom severity is set at 100%, and improvements are represented as a percentage reduction from this baseline. The TNSS encompasses four individual symptoms—nasal blockage, sneezing, nasal pruritus, and rhinorrhea—as well as an aggregated total score.

Both interventions yielded a sustained amelioration in nasal blockage; however, the difference between LNI and SNI was not statistically significant at either the two-week or four-week intervals (*p* = 0.3327 and *p* = 0.1620, respectively; Figure 5A). In the domain of sneezing, LNI demonstrated significant and consistent improvement, a result not mirrored by SNI (*p* = 0.0128 and *p* = 0.0048 at two and four weeks, respectively; Figure 5B). LNI also significantly reduced itchiness of the nose, whereas SNI did not yield significant symptomatic relief (*p* = 0.0067 and *p* = 0.0012 at two and four weeks, respectively; Figure 5C). With respect to rhinorrhea, both treatments were beneficial, but LNI showed a more marked effect after four weeks (*p* = 0.6605 and *p* = 0.0038 at two and four weeks, respectively; Figure 5D).

Considering the overall TNSS, SNI did bring about a reduction in symptoms (from 100% to 82.24% at two weeks and to 79.44% at four weeks), yet LNI’s impact was more substantial (plunging from 100% to 67.89% at two weeks and to 48.95% at four weeks), with these differences achieving statistical significance (*p* = 0.0167 and *p* = 0.0006 at two and four weeks, respectively, as illustrated in Figure 5E).

### 3.5. Secondary Outcomes—Lung Meridian

To evaluate changes in whole-body meridian conductance, we employed the MEAD to assess the 12 meridians in patients with AR before and after treatment with LNI or SNI. Lung MEC was classified into three categories based on a standard conductance range of 30–60 µA: Lung-Low (MEC < 30 µA), Lung-Normal (MEC 30–60 µA), and Lung-High (MEC > 60 µA). We analyzed the changes in lung MEC at baseline, two weeks, and four weeks post-treatment, alongside alterations in TNSS and SNOT-22 scores within these subgroups.

**Figure 6:**
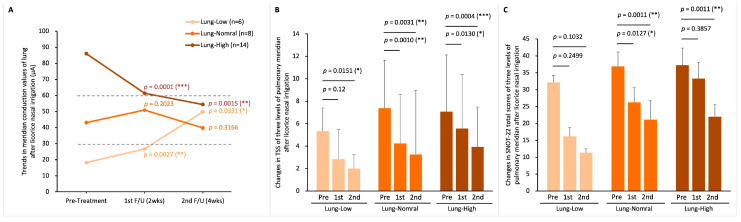
**Lung meridian status before and after LNI treatment.** (**A**) changes in lung MEC after two- and four-weeks treatment in the three MEC-based subgroups: Lung-Low (MEC < 30 µA), Lung-Normal (MEC 30–60 µA), and Lung-High (MEC > 60 µA); (**B**) TNSS variations in the three subgroups; (**C**) total SNOT-22 scores variations in the three subgroups. Within-group changes were analyzed by Student paired *t*-tests. *p*-Values represent comparisons of post-treatment 2 weeks vs. pre-treatment and post-treatment 4 weeks vs. pre-treatment. (* *p* < 0.05; ** *p* < 0.01; *** *p* < 0.0001).

Of the 28 patients in the LNI group, 6 were categorized as Lung-Low, 8 as Lung-Normal, and 14 as Lung-High. Post-LNI treatment, patients in both Lung-Low and Lung-High subgroups showed normalization of lung meridian conductance (MEC before and after LNI therapy in the Lung-Low group: 18.2 ± 6.1 to 49.7 ± 24.2 µA, *p* = 0.0331; in the Lung-Normal group: 43.1 ± 8.1 to 39.7 ± 26.9 µA, *p* = 0.3166; in the Lung-High group: 86.0 ± 23.1 to 54.3 ± 44.7 µA, *p* = 0.0015; Figure 6A). Furthermore, significant improvements in TNSS were observed across all subgroups one month post-treatment (Figure 6B). However, for the SNOT-22 total score, only the Lung-Normal and Lung-High groups exhibited significant improvements, while the Lung-Low group did not show a statistically significant change (Figure 6C).

**Figure 7:**
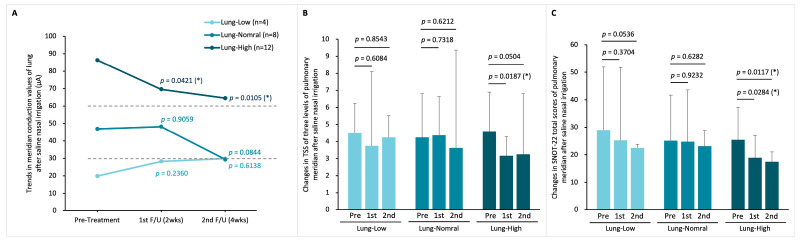
**Lung meridian status before and after SNI treatment.** (**A**) Changes in lung MEC after two- and four-weeks treatment in the three MEC-based subgroups: Lung-Low, Lung-Normal, and Lung-High; (**B**) TNSS variations in the three subgroups; (**C**) total SNOT-22 scores variations in the three subgroups. Within-group changes were analyzed by Student paired *t*-tests. *p*-Values represent comparisons of post-treatment 2 weeks vs. pre-treatment and post-treatment 4 weeks vs. pre-treatment. (* *p* < 0.05).

In the SNI cohort of 24 patients, there were 4 in the Lung-Low, 8 in the Lung-Normal, and 12 in the Lung-High subgroup. One month following SNI treatment, the average value of lung MEC in this group of Lung-Normal was slightly below the lower limit (30 µA) after treatment, but the Lung-Low and Lung-High groups did not show a shift to the normal conductance range, unlike the LNI group (MEC before and after SNI therapy in the Lung-Low group: 19.86 ± 8.05 to 29.84 ± 35.49 µA, *p* = 0.6138; in the Lung-Normal group: 46.89 ± 6.04 to 29.36 ± 23.60 µA, *p* = 0.0844; in the Lung-High group: 86.31 ± 14.81 to 64.50 ± 27.23 µA, *p* = 0.0105; Figure 7A). Additionally, only the Lung-High subgroup demonstrated a statistically significant improvement in TNSS after one month (Figure 7B). Regarding the SNOT-22 score, similar to TNSS, only the Lung-High subgroup showed a significant improvement, with the other subgroups not experiencing notable changes (Figure 7C).

### 3.6. Correlation Analysis Between Changes in Subjective Symptoms and Lung MEC

To explore the association between subjective symptom improvement and objective lung MEC changes, we performed Pearson’s correlation analyses using the change values (Δ = pre − 4-week post) of TNSS, SNOT-22, and lung MEC. Analyses were conducted for all 28 patients and stratified by baseline lung MEC level (Low, Normal, and High MEC).

**Figure 8:**
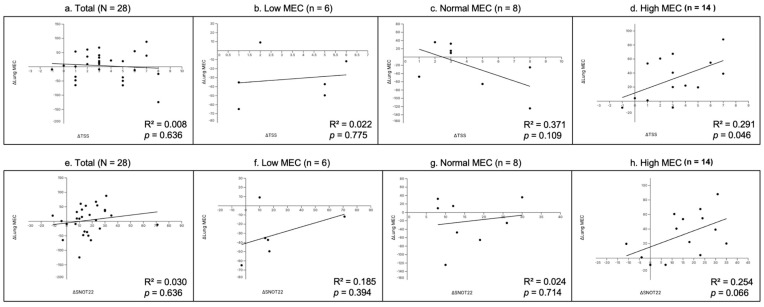
**Correlation analysis between changes in subjective symptom scores and lung MEC.** Each plot shows the relationship between the change in TNSS (ΔTNSS, panels (**a**–**d**)) or SNOT-22 (ΔSNOT-22, panels (**e**–**h**)) and the change in lung MEC (Δ = pre − 4-week post). (**a**,**e**) Total group (N = 28); (**b**,**f**) Low MEC subgroup (n = 6); (**c**,**g**) Normal MEC subgroup (n = 8); (**d**,**h**) High MEC subgroup (n = 12). Solid lines represent linear regression fits. The coefficient of determination (R^2^) and *p*-values are shown on each plot.

As shown in (Figure 8), neither ΔTSS (R^2^ = 0.0087, *p* = 0.636) nor ΔSNOT-22 (R^2^ = 0.030, *p* = 0.375) in the total group exhibited significant correlations with ΔLung MEC. In subgroup analyses, ΔTNSS was weakly correlated in the Low MEC group (R^2^ = 0.0228, *p* = 0.775) but demonstrated a moderate positive correlation that reached significance in the High MEC group (R^2^ = 0.291, *p* = 0.046). ΔSNOT-22 showed weak positive correlations in the Low (R^2^ = 0.185, *p* = 0.394) and Normal (R^2^ = 0.024, *p* = 0.714) MEC groups, and a moderate positive trend in the High MEC group (R^2^ = 0.254, *p* = 0.066).

### 3.7. Adverse Effects

At the follow-up visits, participants were assessed for any adverse sensations associated with nasal irrigation. A small number of participants reported mild ear tingling or a sense of ear fullness during the initial practice of the procedure; however, these symptoms typically resolved within two to three days of continued use. The frequency of ear discomfort did not differ significantly between the two groups.

## 4. Discussion

In a novel integration of TCM and Western clinical practice, this study presents a groundbreaking approach to the management of AR. By objectively quantifying changes in the lung meridian—traditionally associated with respiratory pathologies [21]—both before and after nasal irrigation, we provide the first evidence of meridian alterations in response to therapeutic intervention.

LNI was found to significantly reduce both nasal and extra-nasal symptoms in patients with AR, more so than what was observed with SNI. Notably, patients undergoing LNI experienced substantial improvements in rhinitis-related sleep quality and overall physical and psychosocial well-being. Furthermore, LNI was associated with a normalization of the electrical conductance values along the lung meridian. These significant findings were not paralleled in the SNI group, underscoring the enhanced efficacy of LNI.

Our research marks a significant stride in the objective interpretation and application of TCM meridian theory within a Western medical framework. It establishes a precedent for the successful integration of complementary modalities in the evidence-based treatment of allergic diseases.

TCM posits that diseases manifest through disruptions in the energetic balance of specific meridians and associated organs, presenting as either excess or deficiency in electrical resistance at meridian points. Effective therapeutic interventions, according to TCM, aim to correct these abnormalities, thereby restoring homeostasis [22]. This concept has gained quantitative support through the use of MEAD, which measures electrical conductance at acupoints to objectively assess the state of meridians and their related organs [16]. Our research extends this objective assessment to the realm of nasal irrigation therapies for AR.

In this study, patients with AR presenting with high and normal lung MEC values experienced significant symptomatic improvements in SNOT-22 and TNSS following LNI treatment. Although the low lung MEC subgroup did not show a statistically significant symptomatic change in SNOT-22, post-treatment measurements revealed an increase in their lung MEC to within the normal range, analogous to the high lung MEC group. This indicates that LNI may facilitate positive bioenergetic changes in lung meridian function, which could precede and potentially predict clinical improvement.

GA, a major bioactive compound in licorice, has been shown to modulate immune responses in AR by influencing CD4+ T cells. It adjusts the Th1/Th2 balance and enhances regulatory T cell function as evidenced in cellular assays [23]. In animal studies, GA has been observed to mitigate IgE secretion by B cells and decrease IL-4 levels, thereby promoting a shift towards Th1 dominance and providing an anti-allergic effect [10]. TCM posits that herbal constituents are selectively metabolized and distributed via specific meridians to their target organs [24]. Licorice, according to TCM, is processed by the lung meridian and impacts the respiratory system with anti-inflammatory and immunomodulatory actions [25]. Our findings corroborate the principles of TCM and the observed clinical benefits of LNI for AR. The study highlights that LNI modifies the electrical conductance in the lung meridian, reinforcing the value of nasal irrigation as a treatment for respiratory conditions.

Our additional correlation analysis (Figure 8) provides further insight into the relationship between subjective symptom improvement and objective MEC changes. While no significant overall correlation was observed in the total population, the High MEC subgroup exhibited a moderate and statistically significant positive correlation between ΔTSS and ΔLung MEC, and a similar moderate positive trend between ΔSNOT-22 and ΔLung MEC. These findings suggest that, in patients with higher baseline MEC values, the degree of improvement in subjective symptoms is more closely aligned with objective enhancements. In contrast, the Low and Normal MEC subgroups showed weaker or inconsistent correlations, likely due to smaller sample sizes and greater variability influenced by outliers. Notably, SNOT-22 tended to show stronger correlations with lung MEC changes than TSS, possibly reflecting its broader symptom coverage and sensitivity to treatment-related changes. Future studies with larger cohorts and longer follow-up periods are warranted to validate these findings and clarify the clinical significance of MEC–symptom associations.

Our study has some limitations. First, although there is a statistically significant difference in age between the groups (*p* = 0.037), because the difference is so minimal (37.0 vs. 36.8 years), we believe its impact on the main outcomes in this study is likely negligible. In future studies with larger sample sizes, adjustment for age or sensitivity analyses may be considered. Second, we used SNI as a control instead of a specific placebo that matched the licorice extract solution in color and taste, because we could not find a non-active substance that was similar enough. This means our trial was not blinded, which could lead to biased results, especially in how patients reported their symptoms. To minimize such bias in future studies, a safe, inert substance that closely resembles LE in appearance and taste but lacks anti-inflammatory effects should be developed.

Additionally, stringent controls were established for environmental factors, including ambient temperature and humidity, as well as for the consistency of contact at acupoints and the application of test pressure. Despite these measures, variations in skin moisture and body temperature of the subjects may still introduce an element of variability in the electrodermal resistance measurements, representing a potential bias in the findings [19,24,26]. In sum, while we believe these limitations do not fundamentally undermine the validity of our findings, they warrant cautious interpretation. Future studies should aim for more rigorous blinding, larger sample sizes, adjustment for residual confounders, and replication across settings.

## 5. Conclusions

Nasal irrigation with licorice extract has been demonstrated to significantly alleviate the symptoms of AR more effectively than standard SNI. This includes improvements in both nasal and extra-nasal symptoms, as well as enhancements in sleep quality and mental well-being. Furthermore, LNI has shown promise in normalizing the MEC levels in the lung meridians of patients before and post-treatment, offering objective evidence that supports its efficacy when viewed through the lens of TCM theory. These findings suggest that LNI could serve as a more evidence-based adjunctive treatment for AR patients in future clinical practice.

## Figures and Tables

**Table 1:** The demographic and clinical characteristics of participants.

**Variables**	**LNI**		**SNI**		***p***
	**N = 28**		**N = 24**		
	**n**	**%**	**n**	**%**	
**Gender**					0.121 *
Male	11	39.2	6	25.0	
Female	17	60.7	18	75.0	
**Age** (Year)	37.0 ± 11.7		36.8 ± 9.7		0.037 ^#^
**IgE** (IU/mL)	126.4 ± 142.7		97.6 ± 94.9		0.271 ^#^
**Comorbidities**					
Atopic dermatitis	1	3.5	5	20.8	0.132 *
Asthma	3	10.7	3	12.5	0.815 *
GERD	7	25.0	5	20.8	0.722 *
Hypertension	2	7.14	0	0	-
DM	2	7.14	2	8.33	0.718 *
**Smoking**	0	0	0	0	

Analysis: * Chi-square test or Fisher’s exact test; ^#^ Student *t*-test. Abbreviation: LNI, licorice nasal irrigation; SNI, saline nasal irrigation; GERD, gastroesophageal reflux disease; DM, diabetes mellitus.

**Table 2:** Changes in SNOT-22 scores before and after Licorice or Saline nasal irrigation in rhinitis patients over a month.

SNOT-22 ^a^		Licorice Nasal Irrigation		*p* ^#^	Saline Nasal Irrigation		*p* ^#^
		Pre-Tx ^b^	Post-Tx ^b^		Pre-Tx ^b^	Post-Tx ^b^	
**Nasal symptoms**							
1	Need to blow the nose	2.32 ± 1.12	1.25 ± 0.80	**<0.0001** ***	1.96 ± 1.30	1.04 ± 0.81	**0.0005** **
2	Sneezing	2.29 ± 1.15	1.04 ± 0.88	**<0.0001** ***	1.54 ± 1.06	1.04 ± 0.69	**0.0306** *
3	Runny nose	2.61 ± 0.88	1.36 ± 0.83	**<0.0001** ***	1.79 ± 1.41	1.13 ± 0.80	**0.0065** **
4	Nasal blockage	2.96 ± 1.17	1.43 ± 1.17	**<0.0001** ***	1.58 ± 1.38	0.88 ± 0.95	**0.0021** **
5	decreased sense of taste/smell	1.21 ± 1.32	0.71 ± 1.12	**0.0039** **	0.54 ± 1.22	0.42 ± 1.14	0.1853
6	Thick nasal discharge	1.71 ± 1.27	0.71 ± 1.01	**0.0002** ***	1.38 ± 1.58	0.83 ± 1.34	**0.0394** *
**Extra-nasal symptoms**							
7	Cough	1.54 ± 1.45	0.75 ± 0.84	**0.0014** **	0.88 ± 1.15	0.79 ± 1.18	0.5385
8	Postnasal discharge	2.46 ± 1.10	1.29 ± 1.15	**<0.0001** ***	1.67 ± 1.58	1.04 ± 0.91	**0.0128** *
9	Ear fullness	1.43 ± 1.40	0.89 ± 1.10	**0.0258** *	0.58 ± 0.88	0.63 ± 0.92	0.8326
10	Dizziness	1.29 ± 1.33	0.46 ± 0.74	**0.0021** **	1.04 ± 1.12	0.71 ± 0.95	0.0727
11	Ear pain	1.07 ± 1.21	0.36 ± 0.62	**0.0062** **	0.33 ± 0.70	0.50 ± 0.72	0.2947
12	Facial pain/pressure	0.54 ± 1.04	0.21 ± 0.50	0.0710	0.13 ± 0.34	0.33 ± 0.64	0.1345
**Sleep quality**							
13	Difficulty falling asleep	1.54 ± 1.60	0.96 ± 1.35	**0.0087** **	1.13 ± 1.08	1.04 ± 1.20	0.7036
14	Wake up at night	2.04 ± 1.69	1.46 ± 1.50	0.0578	1.33 ± 1.55	1.38 ± 1.50	0.8798
15	Lack of good night’s sleep	1.71 ± 1.63	1.21 ± 1.45	0.0797	1.50 ± 1.32	1.67 ± 1.49	0.3277
16	Wake up tired	1.96 ± 1.17	1.21 ± 1.17	**0.0077** **	1.92 ± 1.21	1.54 ± 1.32	0.0587
**Physiological status**							
17	Fatigue	1.79 ± 1.23	1.04 ± 0.96	**0.0017** **	1.67 ± 1.27	1.46 ± 1.32	0.3071
18	Reduced productivity	1.46 ± 0.92	0.86 ± 0.97	**0.0103** *	1.00 ± 0.88	0.79 ± 0.78	0.3071
19	Reduced concentration	1.43 ± 0.96	0.79 ± 0.74	**0.0019** **	1.29 ± 1.12	0.92 ± 0.88	0.0951
**Psychosocial status**							
20	Frustrated/restless/irritable	1.11 ± 0.99	0.61 ± 0.63	**0.0079** **	1.33 ± 1.05	1.04 ± 1.46	0.1292
21	Sad	0.71 ± 0.85	0.46 ± 0.58	0.1655	0.75 ± 1.26	0.71 ± 1.30	0.7701
22	Embarrassed	0.89 ± 1.03	0.39 ± 0.57	**0.0079** **	0.58 ± 0.88	0.33 ± 0.56	0.1102
**Total scores**		36.07 ± 15.82	19.46 ± 11.97	**<0.0001** ***	25.92 ± 14.88	20.21 ± 15.43	**0.0065** **

Abbreviations: ^a^ SNOT-22, Sino-Nasal Outcome Test-22; ^b^ Tx, treatment. Statistical Methods: ^#^ paired two-tailed Student’s *t*-test. The use of bold numbers indicates statistical significance. (* *p* < 0.05; ** *p* < 0.01; *** *p* < 0.0001).

## Data Availability

The original contributions presented in this study are included in the article. Further inquiries can be directed to the corresponding author.

## Abbreviations

MEC: meridian electrical conductance; LNI: licorice nasal irrigation; SNI: saline nasal irrigation; TCM: traditional Chinese medicine; TNSS: Total Nasal Symptom Score; SNOT-22: 22-Item Sino-Nasal Outcome Test; MEAD: meridian energy analysis device; HPLC: high-performance liquid chromatography.
